# Microbial Etiology and Antibiotic Resistance Patterns of Urinary Tract Pathogens in Hospitalized Infants in Bahrain: A Tertiary Care Center Experience

**DOI:** 10.7759/cureus.29236

**Published:** 2022-09-16

**Authors:** Deena Mohammed, Hasan M Isa, Maryam F Ali

**Affiliations:** 1 Pediatrics, Salmaniya Medical Complex, Manama, BHR; 2 Pediatrics, Royal College of Surgeons in Ireland - Bahrain, Muharraq, BHR; 3 Pediatrics, Arabian Gulf University, Manama, BHR

**Keywords:** extended-spectrum beta-lactamases, escherichia coli, infants, antibiotic resistance, urinary tract infection

## Abstract

Introduction: Urinary tract infections (UTIs) are fairly common in infants. Early diagnosis and prompt treatment are crucial to avoid complications. However, providing appropriate antibiotic treatment is a challenge to any clinician considering the rise in the urinary pathogens that are resistant to the commonly used antibiotics especially with the emergence of extended-spectrum beta-lactamase (ESBL)-producing organisms. Consequently, this leaves the physician with limited options of antibiotic choices. This study aims to investigate the local resistance patterns of uropathogens causing UTIs in hospitalized infants in Bahrain and to provide guidance for the preferred choice of empirical antibiotic treatment.

Methods: A retrospective cross-sectional study with an analysis of the medical records of infants from birth to one year of age admitted with UTIs at Salmaniya Medical Complex between June 2015 and June 2017 was conducted.

Results: Data were obtained from 117 term infants (no preterm infants were included), with 80 (68.4%) being male. A total of 106 (90.6%) patients were less than six months of age. The most frequently isolated organisms were *Escherichia coli* found in 66 (49.6%) patients, followed by *Klebsiella* in 43 (32.3%). Forty-eight (36%) were ESBL-producing organisms (31 and 17 of *E. coli* and *Klebsiella*, respectively). The highest antibiotic resistance of *E. coli* was found against cephalosporin (55.5%), while penicillin resistance was only 19.4% and co-trimoxazole was 14.4%. Resistance rates of non-*E. coli* organisms were highest against cephalosporin (44.6%), followed by nitrofurantoin (19.1%) and penicillin (18.5%). The overall resistance to cephalosporin antibiotics was highest against cephalothin (a first-generation cephalosporin) (54.9%). The second highest resistance rate was demonstrated against cefuroxime (a second-generation cephalosporin) (50.5%), followed by ceftriaxone (a third-generation cephalosporin) (45.1%). Lower resistance rates were found against cefotaxime (22.3%), followed by ceftazidime (33.3%). A resistance rate of zero was found against cefepime (a fourth-generation cephalosporin). Aminoglycosides and nitrofurantoin demonstrated the lowest resistance rates among all cultured uropathogens. Among all cultures, 3.9% of the *E. coli* were resistant to aminoglycosides, while non-*E. coli* organisms showed zero resistance to aminoglycosides. Regarding the nitrofurantoin antibiotic, 19% of non-*E. coli* bacteria showed resistance, while only 1.7% of *E. coli* were resistant to this antibiotic. Zero resistance to carbapenem was found in *E. coli* organisms (both ESBL and non-ESBL), compared to 1.9% resistance by non-*E. coli* organisms (both ESBL and non-ESBL combined).

Conclusion: *E. coli* is the predominant uropathogen causing UTI in infants with high rates of ESBL organisms in our community. Since the highest resistance was found against cephalosporins, aminoglycosides and nitrofurantoin are the preferred empirical antibiotic options for the treatment of UTIs. For infants with ESBL organisms, carbapenem antibiotics represent a good treatment option.

## Introduction

Urinary tract infections (UTIs) are fairly common in infants. The estimated overall prevalence is 7% in infants and young children; however, it varies between countries according to different factors such as age, gender, race, and circumcision status among males [1].

Providing appropriate and adequate treatment is important to prevent potential permanent complications such as kidney scarring, hypertension, and chronic kidney disease [1,2]. Moreover, once a febrile UTI is suspected, the early empirical initiation of antimicrobial therapy is crucial to halt the formation of new scarring. A delay in treatment of two days or more can significantly increase the probability of new kidney scarring by approximately 47% [3]. The choice of the empirical antibiotic treatment of UTIs invariably depends on commonly encountered pathogens and antibiotic resistance patterns in local communities. *Escherichia coli* is well known to be the most common causative organism for UTIs in children worldwide. Likewise, a recent study from Bahrain found that *E. coli* was the most common uropathogen causing UTI in infants, accounting for 50% of cases, followed by *Klebsiella pneumoniae* (32%) [4]. These findings are very similar to studies published from countries neighboring Bahrain [5-8]. Moreover, the study from Bahrain also documented a high rate of extended-spectrum beta-lactamase (ESBL)-producing organisms (43.4% of *E. coli* bacteria and 31.8% of *K. pneumoniae* infections) [4]. The rising incidence of ESBL organisms is an emerging problem worldwide and is considered a major health threat, which could be due to unjustified, widespread, and inappropriate use of antibiotics. Other potential risk factors for the high ESBL prevalence include the presence of underlying renal anomalies, clean intermittent catheterization (CIC), and previous hospitalization necessitating the use of antibiotics [9-12].

The best choices for empirical antibiotics for UTIs in infants and their resistance patterns were reported in several studies worldwide, which vary between countries [13-15]. However, the resistance pattern was not documented for infants with UTIs in Bahrain. The current study aims to investigate the local resistance patterns of uropathogens causing UTIs in hospitalized infants in Bahrain and to provide guidance for the preferred choice of empirical antibiotic treatment.

## Materials and methods

Study design and data collection

A retrospective cross-sectional study with an analysis of medical records of all infants from birth to one year of age admitted with UTIs in the Pediatric Department, Salmaniya Medical Complex, Bahrain between June 2015 and June 2017 was conducted. Data were collected from patients' charts and electronic medical records. Demographic data including age at the time of presentation, gender, nationality, birth weight, presence of underlying urological anomaly, and clinical presentation were gathered, along with data about the method of urine collection and laboratory results. The results of urine cultures, the type of empirical antibiotics used, and the sensitivity patterns of different pathogenic organisms were reviewed.

Inclusion and exclusion criteria

We included infants from birth to 12 months of age. All infants were full term, and no preterm infants were included. Accepted urine samples were limited to those collected by transurethral bladder catheterization or suprapubic aspiration. Samples collected by bag and those showing mixed growth of organisms indicating possible contamination of the sample were excluded. UTI was defined as “the presence of 10,000 to 50,000 colony-forming units (CFU) per mL” [16].

Statistical analysis

Data were statistically analyzed using SPSS version 24 (IBM Corp., Armonk, NY). Categorical variables were presented as numbers and frequencies. Numerical variables were presented as a mean and standard deviation (SD) or median and interquartile range as appropriate. The different pathogenic organisms were divided into two groups: *E. coli* and non-*E. coli*. These two groups were compared in terms of antibiotic resistance using a chi-squared test. P-values < 0.05 were considered statistically significant.

Ethical statement

This research was reviewed and approved by the Research Committee for Government Hospitals in Bahrain (IRB number: 53160522).

## Results

During the study period, a total of 125 patients were initially studied, and eight patients were excluded due to the non-availability of sensitivity reports for the cultured urine organisms. Consequently, 117 (93.6%) patients who had at least one report of antibiotic sensitivity were included in the study. Demographic data of the included patients are shown in Table 1. Most of these infants were admitted with their first UTI episode and were not known to have an underlying urological anomaly or renal disease; however, further investigations by renal ultrasound and micturating cystourethrogram (MCUG) revealed that 29 (23.2%) had urological anomalies.

**Table 1:** Types of organisms cultured in children with urinary tract infection * Values presented as numbers (%).

Variables	n (%)^*^
Gender	
Females	37 (31.6)
Males	80 (68.4)
Age at time of presentation	
0-6 months	106 (90.6)
6-12 months	8.0 (6.8)
>12 months	3.0 (2.6)
Nationality	
Bahraini	96 (82.1)
Non-Bahraini	21 (17.9)

The median number of antibiotic sensitivity tests was nine (interquartile range (IQR) = 8-11) tests per patient. Eighty (68.4%) patients were male. A total of 106 (90.6%) patients were less than six months of age at the time of presentation, with a median age of one month (IQR = 0.28-2.5 months). The majority of patients (96, 82.1%) were Bahraini. Of the remaining 21 (17.9%) non-Bahraini patients, four patients were from India, two each were from Pakistan, Bangladesh, and Egypt, and one each was from Saudi Arabia, Philippines, Sri Lanka, and Syria, while the remaining seven patients were from unspecified countries.

Different types of microorganisms cultured from the urine samples are shown in Table 2. A total of 10 organisms were isolated from 133 positive urine sample cultures. The most frequently isolated organism was *E. coli*, which was found in 66 (49.6%) urine samples, followed by *Klebsiella* in 43 (32.3%) samples. ESBL-producing organisms were seen in 48 (36%) samples (31 of *E. coli* type and 17 of *Klebsiella*). Sixteen (12%) patients had two organisms cultured. The most frequent organism combinations were *Klebsiella* and *E. coli*, followed by *E. coli* and *Enterococcus*. Sixteen patients had another episode of UTI that has been included in the result; however, as shown in Table 2, the results of these episodes were separated in order not to affect the overall prevalence of uropathogens, despite that *E. coli* remained the commonest uropathogen followed by *Klebsiella*. Even after excluding the 16 samples, the prevalence of both *E. coli *and *Klebsiella* increased from 49.6% and 32.3% to 52.1% and 34.1%, respectively. These additional sixteen urine samples have been taken during different UTI episodes and were included in the sensitivity analysis as they should not affect the resistance pattern.

**Table 2:** Demographic data of 117 patients with positive urine cultures * Values presented as numbers (%).

Type of organism	Microorganism 1, n (%)^*^	Microorganism 2, n (%)^*^	Total, n (%)^*^
Escherichia coli	61 (52.1)	5.0 (4.3)	66 (49.6)
Klebsiella	40 (34.1)	3.0 (2.5)	43 (32.3)
Enterococcus	5.0 (4.3)	6.0 (5.1)	11 (8.2)
Pseudomonas aeruginosa	4.0 (3.4)	0.0 (0.0)	4.0 (3.0)
Enterobacter	2.0 (1.7)	1.0 (0.9)	3.0 (2.2)
Stenotrophomonas maltophilia	2.0 (1.7)	0.0 (0.0)	2.0 (1.5)
Proteus mirabilis	1.0 (0.9)	0.0 (0.0)	1.0 (0.7)
Staphylococcus aureus	1.0 (0.9)	0.0 (0.0)	1.0 (0.7)
Staphylococcus epidermidis	1.0 (0.9)	0.0 (0.0)	1.0 (0.7)
Streptococcus agalactiae	0.0 (0.0)	1.0 (0.9)	1.0 (0.7)
Total	117 (87.9)	16 (12)	133 (100)

The non-Bahraini infants had a higher percentage of *E. coli* organisms compared to Bahraini infants (n = 13/21 (61.9%) vs. n = 48/96 (50%), respectively), but this difference was not statistically significant (p = 0.323); moreover, the non-Bahraini infants had a higher percentage of ESBL organisms compared to Bahraini infants (n = 11/21 (52.4%) vs. n = 35/96 (36.5%), respectively), but again this difference was not statistically significant (p = 0.176).

Antibiotic resistance against *E. coli* and *non-E. coli* organisms is shown in Table 3. The highest resistance rates for both *E. coli* and non-*E. coli* organisms were of cephalosporins (55.5 and 44.6, respectively), yet the difference between the two groups of organisms in terms of resistance to antibiotics was not statistically significant (p = 0.945). The overall resistance to cephalosporin antibiotics was highest against cephalothin (a first-generation cephalosporin) (61 (54.9%) samples out of 111 positive cultures). The second highest resistance rate was demonstrated against cefuroxime (a second-generation cephalosporin) (55 (50.5%) out of 109 cultures), followed by ceftriaxone (a third-generation cephalosporin) (50 (45.1%) out of 109 cultures). Lower resistance rates were found against cefotaxime (a third-generation cephalosporin) (two (22.3%) out of nine positive cultures), followed by ceftazidime (two (33.3%) out of six positive cultures). A resistance rate of zero was found against cefepime (a fourth-generation cephalosporin) (tested on four positive cultures).

**Table 3:** Reported antibiotics resistance against Escherichia coli and non-Escherichia coli organisms * Values presented as numbers (%). ^†^ P-values < 0.05 were statistically significant.

Type of antibiotics	*Escherichia coli*, n (%)^*^ (n = 180)	Non-*Escherichia coli *organisms, n (%)^*^ (n = 157)	P-value^†^
Cephalosporin	100 (55.5)	70 (44.6)	0.945
Penicillin	35 (19.4)	29 (18.5)	0.467
Co-trimoxazole	26 (14.4)	18 (11.5)	0.471
Fluoroquinolone	8.0 (4.4)	7.0 (4.4)	0.784
Aminoglycosides	7.0 (3.9)	0.0 (0.0)	0.015
Nitrofurantoin	3.0 (1.7)	30 (19.1)	<0.001
Glycylcyclines	1.0 (1.25)	0.0 (0.0)	0.468
Carbapenem	0.0 (0.0)	3.0 (1.9)	0.213

Compared to *E. coli* organisms, the non-*E. coli* organisms had a statistically significant lower rate of resistance to aminoglycosides (p = 0.015). On the other hand, the *E. coli* organisms were significantly more sensitive to nitrofurantoin compared to non-*E. coli *organisms (p < 0.001).

The antibiotic resistance of ESBL organisms is shown in Table 4. All ESBL organisms showed resistance to all tested cephalosporins (ceftriaxone, cephalothin, cefuroxime, and cefotaxime) and all tested penicillin antibiotics, which included both penicillin combined with a beta-lactamase inhibitor (amoxicillin-clavulanate and penicillin alone as ampicillin). Six (14.35) of the 42 cultured ESBL organisms were resistant to gentamicin, while none were resistant to carbapenems or tigecycline (tested in 46 and 45 cultures, respectively).

**Table 4:** Reported antibiotics resistance against 48 cultured ESBL-producing organisms * Values presented as numbers (%). ESBL: extended-spectrum beta-lactamase.

Type of antibiotics	Resistance, n (%)^*^	Total tested
Ceftriaxone	46 (100)	46 (95.8)
Cephalothin	46 (100)	46 (95.8)
Cefuroxime	44 (100)	44 (91.7)
Cefotaxime	2.0 (100)	2.0 (4.2)
Amoxicillin-clavulanate	46 (100)	46 (95.8)
Ampicillin	3.0 (100)	3.0 (6.3)
Co-trimoxazole	35 (76)	46 (95.8)
Nitrofurantoin	14 (31.1)	45 (93.7)
Norfloxacin	12 (25)	47 (97.9)
Gentamicin	6.0 (14.3)	42 (87.5)
Carbapenem	0.0 (0.0)	46 (95.8)
Tigecycline	0.0 (0.0)	45 (93.7)

## Discussion

This study showed that gram-negative organisms remained the most frequently isolated uropathogens causing UTIs, with *E. coli* accounting for most of the cases (49.6%) and *K. pneumoniae* standing as the second most common organism (accounting for 32.3%). These results are similar to those reported in other studies in our region. Husain et al. from Kuwait showed that *E. coli* was responsible for 69% of UTIs in children, followed by *K. pneumoniae* (11.4%) [6]. In Saudi Arabia, Garout et al. also found that *E. coli* was the causative organism in 41.2% of UTIs, followed by *K. pneumoniae* (19.6%) [7]. Additionally, Sharef et al. from Oman showed that *E. coli *was accountable for 69% of UTIs, followed by *K. pneumoniae* (17%) [8].

Our study illustrated a strikingly high rate of ESBL-producing organisms seen in 48 (36%) samples (31 positive samples for *E. coli* and 17 for *Klebsiella*). This is much higher than what was reported in a recent regional study in Saudi Arabia by Hameed et al., which showed 16 (7.9%) ESBL uropathogens among 202 children [5]. In Oman, a 10% ESBL rate was reported among 175 children [8]. Moreover, in Greece, Vazouras et al. also reported a very low incidence of ESBL uropathogens (1.7%) among 230 children with UTIs [13]. The studies mentioned are comparable to our study as they deal with UTIs in children including infants; the population from Kuwait, Oman, and Saudi Arabia are comparable to our population; however, the population in Greece varies in ethnicity. Regarding the methodology, all of these studies were retrospective cross analyses similar to our study.

The high rate of ESBL-producing organisms and antibiotic resistance in this study, which was conducted in the main tertiary center in Bahrain with referrals from all over the country, is alarming and in keeping with the global challenge of the rising antibiotic resistance. Lately, antibiotic resistance has become an evolving global phenomenon complicating the treatment of UTIs. A recent systemic review and meta-analysis established high rates of antibiotic resistance in pediatric UTIs caused by *E. coli* in a primary care setting [17]. Multifactorial causes are responsible for this growing problem worldwide; mainly the inappropriate, unjustified, and overuse of antibiotics.

In the present study, the highest antibiotic resistance was found against cephalosporin (55.5% for *E. coli* and 44.6% for non-*E. coli*). Resistance to the cephalosporin antibiotic group was highest against cephalothin (a first-generation cephalosporin), followed by cefuroxime (a second-generation cephalosporin) and ceftriaxone (a third-generation cephalosporin), all of which are extensively used in local practice, especially in the primary care and private sector. On the other hand, among the third-generation cephalosporins, cefotaxime, which is commonly prescribed for inpatient treatment of UTI, showed only 22.3% resistance, which still makes it a reasonable choice for empirical treatment. Likewise, in Saudi Arabia, Hameed et al. showed a very low resistance rate of 4% against cefotaxime [5]. The lowest resistance rates were demonstrated against the fourth-generation cephalosporin (cefepime, 0%).

Broad-spectrum penicillin is commonly used as an empirical antibiotic for UTIs in children. However, in recent years, there has been a drastic increase in resistance rates against penicillin reported all over the world. In the current study, penicillin resistance was found in only 19.4% of cases, while resistance to co-trimoxazole was found in 14.4%. However, Hameed et al. from Saudi Arabia recently reported higher resistance to ampicillin (68%) and co-trimoxazole (54%) compared to our findings [5]. Moreover, Demir et al. in Turkey studied 842 children admitted with UTIs and showed that the highest resistance rates of all cultured microorganisms were seen against ampicillin (87.3%), followed by cefuroxime (71.6%) [18].

The low penicillin resistance rates in the current study, as well as the high resistance to cephalosporin, could be a reflection of the local physicians’ clinical practices, as they have almost abandoned the use of penicillin, while they extensively overuse cephalosporins.

Aminoglycosides and nitrofurantoin demonstrated the lowest resistance rates among all cultured uropathogens. Among all cultures, 3.9% of the *E. coli* were resistant to aminoglycosides, while non-*E. coli* organisms showed zero resistance to aminoglycosides.

Regarding the nitrofurantoin antibiotic, 19% of non-*E. coli* bacteria showed resistance, while only 1.7% of *E. coli* were resistant to this antibiotic. This finding supports the empirical use of oral nitrofurantoin as a preferred oral antibiotic treatment for UTIs. However, this might be limited by the high prevalence of glucose-6-phosphate dehydrogenase (G6PD) deficiency among the local population in Bahrain (22.3%) [19].

Finally, the *E. coli* cultures in our study exhibited zero resistance to the carbapenem antibiotics group, including ESBL-producing *E. coli*. Non-*E. coli* organisms also showed low resistance to carbapenem (1.9%).

## Conclusions

*E. coli* is the predominant uropathogen causing UTIs in infants, with a high rate of ESBL organisms existing in our community. Since the highest resistance was found against cephalosporins, aminoglycosides and nitrofurantoin are the preferred empirical antibiotic options for the treatment of UTIs. For infants with ESBL organisms, carbapenem antibiotics represent a good treatment option.

Our study represents the first report from Bahrain tackling the prevalence and antibiotic resistance patterns of uropathogens in community-acquired UTIs in hospitalized infants. However, being a retrospective study, a lack of some demographic data is expected. Moreover, the relatively small sample size might have imposed another limitation. Despite its limitations, this study sheds light on an important topic in pediatric clinical practice and will help improve clinical practices and antibiotic stewardship.
